# Encoding of cutaneous stimuli by lamina I projection neurons

**DOI:** 10.1097/j.pain.0000000000002226

**Published:** 2021-03-24

**Authors:** Kim I. Chisholm, Laure Lo Re, Erika Polgár, Maria Gutierrez-Mecinas, Andrew J. Todd, Stephen B. McMahon

**Affiliations:** a Neurorestoration Group, Wolfson Centre for Age-Related Diseases, King's College London, London, United Kingdom; b Spinal Cord Group, Institute of Neuroscience and Psychology, College of Medical, Veterinary and Life Sciences, University of Glasgow, Glasgow, United Kingdom

**Keywords:** Confocal, Genetically encoded calcium indicators, Spinal cord, Dorsal horn, Thermal sensation, Nociception, Pain

## Abstract

Lamina I of the dorsal horn, together with its main output pathway, lamina I projection neurons, has long been implicated in the processing of nociceptive stimuli, as well as the development of chronic pain conditions. However, the study of lamina I projection neurons is hampered by technical challenges, including the low throughput and selection biases of traditional electrophysiological techniques. Here we report on a technique that uses anatomical labelling strategies and in vivo imaging to simultaneously study a network of lamina I projection neurons in response to electrical and natural stimuli. Although we were able to confirm the nociceptive involvement of this group of cells, we also describe an unexpected preference for innocuous cooling stimuli. We were able to characterize the thermal responsiveness of these cells in detail and found cooling responses decline when exposed to stable cold temperatures maintained for more than a few seconds, as well as to encode the intensity of the end temperature, while heating responses showed an unexpected reliance on adaptation temperatures.

## Abstract 2

Supplemental Digital Content is Available in the Text.

We here report on the use of in vivo optical imaging to study lamina I projection neurons and their response to cutaneous stimuli.

## 1. Introduction

Lamina I of the dorsal horn has long been known to be a site of integration of nociceptive and thermal primary afferent information.^53^ Despite this nociceptive specificity attributed to lamina I, it is a layer of highly heterogenous neuronal populations, consisting primarily of various types of interneurons.^52^ Within lamina I, however, there is also a small population of projection neurons. Despite their low number, these neurons provide a main output pathway for information integrated in the superficial dorsal horn. Thus, they have been implicated not only in nociception and thermoreception^2–4,16,17,24,29,33,34,55,56,62^ but importantly also in the development of chronic pain conditions.^25,37,43,51^ Interestingly, ablation studies, using substance P saporin conjugates, have concluded that lamina I projection neurons, despite being sensitive to noxious and thermal stimuli also in the naive state, are not essential for normal pain processing. Instead, the loss of lamina I projection neurons only becomes noticeable in a pathological context, eg, during neuropathic or inflammatory pain.^37,43^ A loss of this population of neurons led to an attenuation of the disease phenotype, strongly implicating lamina I projection neurons in the development of chronic pain.

However, studying the physiology of these cells is challenging because they make up a small percentage of cells in lamina I (eg, only 5% of lamina I cells in the mouse are spinoparabrachial projection neurons^5^). Therefore, studies that do not distinguish between interneurons and projection neurons are unlikely to provide a clear picture of projection neuron physiology.^5,49^ Instead retrograde labelling or antidromic stimulation need to be used to isolate projection neurons, which, by definition, are the only cells selected by such techniques. Although original studies conducted in rodents, cats, and nonhuman primates suggest that thalamic nuclei provide an important target for lamina I projection neurons,^6,13,16,17,48,61,62^ it is now known that 85% to 95% of lamina I projection neurons in the rat lumbar dorsal horn project through the dorsolateral funiculus of the spinal cord (and not the ventrolateral white matter) to the lateral parabrachial nucleus.^38,44,49,53^ It is also known that 80% to 97% of lamina I neurons projecting to the thalamus also project to the parabrachial nucleus in the rat,^1,23^ suggesting that although lamina I projection neurons terminate in multiple brain areas,^1,23,30,39,49,54^ spinoparabrachial projection neurons represent a substantial proportion of all lamina I projection neurons.^5,49^

We have therefore used anatomical labelling techniques to selectively study lamina I projection neurons by injecting adeno-associated virus (AAV) encoding the calcium indicator GCaMP6s into the lateral parabrachial nucleus. Combining such labelling techniques with standard confocal microscopy allowed us to study the function of this spinal cord output pathway in the intact animals in response to electrical and natural stimuli. We were able to confirm the nociceptive specificity of this pathway using in vivo imaging and found an additional and unexpected sensitivity of this system to innocuous cooling stimuli.

## 2. Materials and Methods

Both male and female adult C57BL/6J mice (Charles RiverLaboratories, Wilmington, MA) were used for all experiments. No obvious differences were noted between male and female mice and therefore the data was pooled. Mice were housed on a 12/12 hours light/dark cycle with a maximum of 5 animals per cage. Food and water were available ad libitum. All experiments were performed in accordance with the United Kingdom Home Office Animals (Scientific Procedures) Act (1986). Animal numbers for each experiment are noted in the appropriate figure legends.

### 2.1. Administration of adeno-associated virus 9-GCaMP6s into the lateral parabrachial area

Sterile technique was maintained throughout the surgery and mice maintained at around 37°C core body temperature using a homeothermic heating mat with a rectal probe (Frederick Haer Company, Inc., Bowdoin, ME). Isoflurane (in oxygen, 5% for induction and 2% for maintenance) was used to anaesthetise mice to a surgical depth. After anaesthetic induction, Carprieve (0.025 mg; Norbrook Laboratories, Newry, United Kingdom) and sterile normal saline (0.5 mL at 0.9%) were administered subcutaneously for postsurgical analgesia and rehydration, respectively. Eye gel was applied to moisten and protect eyes (Viscotears, Liquid Gel, Novartis).

Mice were placed into a stereotaxic frame, and a single incision was made into the scalp to expose the underlying skull. Using bregma and lambda as landmarks to locate the lateral parabrachial area, a small hole was drilled through the skull using a dental drill (Ideal Micro-Drill, WPI). Using a microinjection setup with a glass pipette, a single injection of 800 nL of AAV9.CAG.GCaMP6s.WPRE.SV40 (UPENN Vector Core, AV-1-PV2833, 1.1 × 10^13^ gc/mL) was infused into the right lateral parabrachial area at a rate of 100 nL/minute. The glass pipette remained in place for a further 2 to 5 minutes to allow any residual liquid bolus to diffuse, before being slowly withdrawn. The skin incision was closed, and mice recovered for a period of at least 5 days, during which expression of GCaMP6s in spinoparabrachial neurons occurred.

### 2.2. In vivo imaging of lamina I projection neurons

After recovery and GCaMP expression, mice were reanaesthetised using urethane (12.5% wt/vol). An initial dose of 0.3 mL (37.5 mg urethane) was given intraperitoneal. After partial anaesthesia, mice were placed on a homeothermic heating mat with a rectal probe (Frederick Haer Company, Inc.) to control core body temperature around 37°C. Further doses of urethane were administered at ∼15-minute intervals, depending on reflex activity. Surgical depth was achieved when no hind limb or corneal reflexes were observed. To facilitate smooth breathing and reduce breathing-related movement, a tracheal catheter was installed while mice were breathing spontaneously. For hydration, 0.5 mL of sterile normal saline (0.9%) was administered subcutaneously.

To expose the dorsal horn of the spinal cord, the hair was removed from the back of the mice and a single skin incision was made over the lumbar enlargement. The muscle and connective tissue overlying the lumbar enlargement of the spinal cord were carefully removed and a laminectomy performed using rongeurs. The dura mater was washed and moistened with normal saline. The exposed spinal segment was stabilised in a slightly lateral recumbent orientation (to optimise access to the left dorsal horn) using spinal clamps, fastened on the intact vertebrae on either side of the exposure. The exposed segment with intact dura was covered with silicone elastomers (World Precision Instruments, Ltd, Hitchin, United Kingdom) to maintain a physiological environment and prevent drying of the cord.

To acquire time-lapse recordings of the dorsal horn, the mice were placed under the Eclipse Ni-E FN upright confocal/multiphoton microscope (Nikon, Melville, NY), with ambient temperature maintained at 32°C. A 488-nm argon ion laser line was used to acquire images through a 10× dry objective and a fully opened pinhole. A 500- to 550-nm bandpass filter was used for signal collection, and images were collected at 0.5 to 4 Hz (depending on the strength of the signal but typically this was at 4 Hz), with a resolution of 512 × 256 pixels.

### 2.3. Activation of projection neurons with electrical stimuli

To visualise the response of projection neurons to electrical stimulation, a subset of mice were stimulated directly through the sciatic nerve. To achieve this, the left sciatic nerve (ipsilateral to the imaged dorsal horn) was exposed: a small incision in the shaved skin of the leg, just medial of the femur, exposed the underlying muscle that was blunt dissected to expose the sciatic nerve. A custom-made cuff electrode with Teflon-insulated silver wire (Ø 0.125 mm; Advent Research Materials, Oxford, United Kingdom) was positioned underneath the sciatic nerve while partially enveloping it on either side. To isolate the sciatic nerve and electrode and to prevent drying, the preparation was covered with dental silicon impression compound (Heraeus Kulzer, Basingstoke, United Kingdom). Electrical stimuli were delivered through a biphasic stimulator (World Precision Instruments), which delivered trains of square wave pulses. Stimulation parameters of 50 µs and 100 µA were used to activate Aβ fibres, 250 µs and 250 µA for Aδ fibres, and 1 ms and 5 mA stimuli were used for suprathreshold stimulation that was expected to activate all fibres, including both myelinated and unmyelinated axons.

### 2.4. Activation of projection neurons with mechanical stimuli

Mechanical stimuli were applied to the left paw (ipsilateral to the recording side) and consisted of brushing the plantar surface (in a medial to lateral direction) or pinching across the entire surface of the paw, using blunt forceps. To maintain consistency across experimental preparations, an effort was made to apply the stimuli to similar areas across each mouse paw.

### 2.5. Activation of projection neurons with thermal stimuli

To apply controlled thermal stimuli to the periphery of imaged mice, a Peltier device (TSAII, Medoc, Ramat Yishay, Israel) was used. A 16 × 16-mm probe was securely placed onto the plantar surface of the left hind paw. The temperature of the block was increased to up to 50°C and decreased down to 4°C. A temperature of 32°C was used as a standard skin temperature. A series of ramp and hold stimuli were used. Four sets of heating ramps and 4 sets of cooling ramps were applied as described below.

### 2.6. Heating ramps

#### 2.6.1. Simple heating ramps

Nine consecutive increments occurred as steps of 2 from 32°C to maximum 50°C (ie, 32 to 34°C, 32 to 36°C … 32 to 50°C). Temperature increases occurred at 2°C/s and returns to baseline at 4°C/s, and the holding temperature was maintained for 5 seconds. The baseline temperature of 32°C was maintained for 90 seconds between each increment.

#### 2.6.2. Rate of change heating ramps

Three consecutive increments occurred as steps from 32°C to 50°C. The rate of temperature increase varied from slow (0.2°C/s) to medium (0.5°C/s) to fast (2°C/s). The temperature returned to baseline at 8°C/s. The baseline temperature of 32°C was maintained for 90 seconds between each ramp.

#### 2.6.3. Baseline variable heating ramps

Five consecutive increments occurred as increases of 13°C from different baseline temperatures. These temperature ranges were chosen to fit the ranges of stimulation from just below skin temperature to 50°. The baseline temperature was varied from 22 to 37°C (22, 27, 32, 34, and 37°C), and end temperatures were varied commensurately from 35 to 50°C (35, 40, 45, 47, and 50°C). Temperature increases occurred at 3.2°C/s and returns to baseline at 8°C/s, and the holding temperature was maintained for 5 seconds. The baseline temperatures were maintained for 2 minutes before each ramp.

#### 2.6.4. End temperature stable heating ramps

Five consecutive increments occurred as increases from variable baseline temperatures up to 50°C. The baseline temperature was varied from 22 to 42°C in steps of 5°C (22, 27, 32, 37, and 42°C). Temperature increases occurred at variable rates to maintain the total stimulation period stable (ranging from 2 to 7°C/s). Returns to baseline occurred at 8°C/s, and the holding temperature was maintained for 5 seconds. The baseline temperatures were maintained for 2 minutes before each ramp.

### 2.7. Cooling ramps

#### 2.7.1. Simple cooling ramps

Six consecutive decremental steps occurred from 32°C to minimum 4°C (32 to 27°C, 32 to 22°C, 32 to 17°C, 32 to 12°C, 32 to 7°C, and 32 to 4°C). Temperature decreases occurred at 2°C/s and returns to baseline at 4°C/s, and the holding temperature was maintained for 5 seconds. The baseline temperature of 32°C was maintained for 90 seconds between each decremental step.

#### 2.7.2. Rate of change cooling ramps

Three consecutive decremental steps occurred as steps from 32 to 10°C. The rate of temperature decrease varied from slow (0.2°C/s) to medium (0.5°C/s) to fast (2°C/s). The temperature returned to baseline at 8°C/s. The baseline temperature of 32°C was maintained for 90 seconds between each ramp.

#### 2.7.3. Baseline variable cooling ramps

Six consecutive decremental steps occurred as decreases of 10°C from different baseline temperatures. The baseline temperature was varied from 17 to 42°C in steps of 5°C (ie, 17, 22, 27, 32, 37, and 42°C), and end temperatures were varied commensurately from 7 to 32°C (7, 12, 17, 22, 27, and 32°C). Temperature decreases occurred at 2.5°C/s and returns to baseline at 8°C/s, and the holding temperature was maintained for 5 seconds. The baseline temperatures were maintained for 2 minutes before each ramp.

#### 2.7.4. End temperature stable cooling ramps

Six consecutive decremental steps occurred as decreases from variable baseline temperatures down to 10°C. The baseline temperature was varied from 17 to 42°C in steps of 5°C (17, 22, 27, 32, 37, and 42°C). Temperature decreases occurred at variable rates to maintain the total stimulation period stable (ranging from 1.7 to 8°C/s). Returns to baseline occurred at 8°C/s, and the holding temperature was maintained for 5 seconds. The baseline temperatures were maintained for 2 minutes before each ramp.

Each ramp is graphically represented in the appropriate figure.

### 2.8. Experimental design and statistical analysis

The NIS (Nikon Imaging Software) Elements AR 0.30.01 (Nikon, align application) was used to correct drift and movement in time-lapse recording. If movement issues remained or were exacerbated, the manual drift correction plugin (Fiji/ImageJ) was used. Additional processing of images was performed using Fiji/ImageJ version 1.48v. A combination of Microsoft Office Excel 2013, IBM SPSS Statistics 23 package, and RStudio 0.99.893 were used for statistical analysis and graphical display of data. All statistical tests and sample sizes are noted in the associated figure legends.

For analysis and graphing purposes, normalised traces of calcium fluorescence were generated. To achieve this, regions of interest (ROIs) were drawn around individual cell bodies and the pixel intensity over the area averaged for each image in a time-lapse recording. In addition, a region of background was drawn over the same period and its signal was subtracted from each individual ROI. This background subtracted signal was then normalised using the following formula:

$$\frac{\Delta \text{F}}{\text{F}}=\frac{{\text{F}}_{\text{t}}-{\text{F}}_{0}}{{\text{F}}_{0}}$$

Where F_t_ defines the average fluorescence of each ROI at time t and F_0_ denotes the average fluorescence of each ROI during a baseline recording period, before the commencement of any stimulation. In this article, ΔF/F is expressed as a percentage.

To determine a positive response, a threshold of 70% above baseline fluorescence plus 4 SD of the fluorescence across the baseline period was set. This threshold was based on comparisons with visual observations (by a trained investigator) of positive responses, to maximise true positives and minimise false positives.

It should be noted that the baseline period is considered independently for each stimulus applied, instead of at the beginning of each experiment. For this purpose, a period of 5 seconds, starting 10 seconds before each stimulus, is considered the baseline period to assess the response cut-off. This is to avoid positive responses being evaluated from signal drift.

It was noted that postmortem cells accumulated calcium and therefore increased their fluorescence intensity. When appropriate the postmortem cell count was therefore used to calculate the percentage of responding cells.

## 3. Results

### 3.1. Projection neurons can be visualised in the dorsal horn in vivo

**Figure 1.**
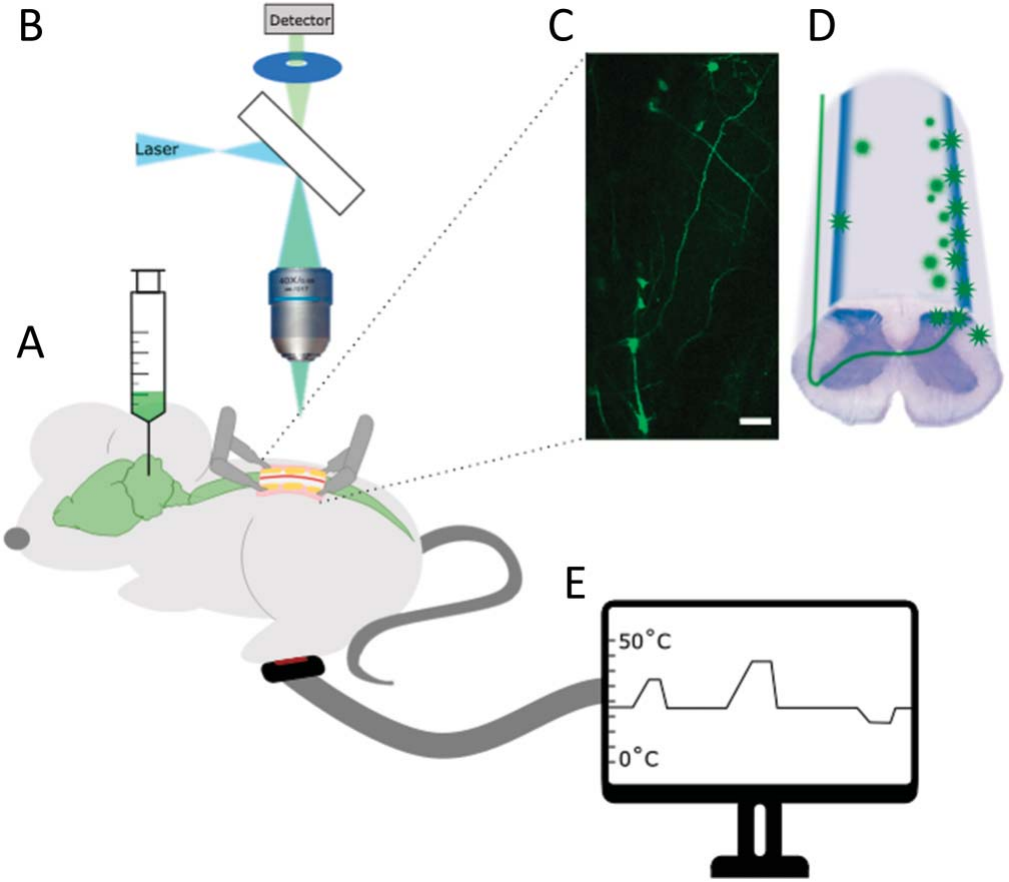
Description of experimental setup. (A) Mice were injected with AAV9 expressing GCaMP at least 5 days before imaging. (B) Mice were imaged with a one-photon microscope and (C) labelled cells visualised in the dorsal horn of the spinal cord. Scale bar = 100 μm. (D) Cartoon of cellular labelling after parabrachial injections. During imaging sessions the ipsilateral peripheral paw of the mouse was stimulated electrically (with a cuff electrode around the sciatic nerve), mechanically (through brush or pinch), or (E) thermally (using a Peltier device applied to the plantar surface of the paw). AAV, adeno-associated virus.

To visualise the function of projection neurons in situ in the spinal cord, mice were injected with AAV9 expressing GCaMP6s, unilaterally into the lateral parabrachial nucleus. A single injection efficiently back labelled a plexus of lamina I projection neurons (in line with previous findings^5^) (Figs. 1A–D). The labelling was strong enough to visualise the cell bodies and processes of many projection neurons simultaneously (Fig. 1C). Using a custom-made stage, it was possible to sufficiently stabilise the vertebral column to visualise projection neurons in situ using standard confocal microscopy (Figs. 1B and C). To provide a greater depth of view, the pinhole of the microscope was maintained open. This reduced the effect of the natural curvature of the spine and any biological movements, which would cause a loss of focus.

To study the function of projection neurons, various stimuli were applied to the ipsilateral paw, including electrical, mechanical, and thermal stimuli (Fig. 1E).

### 3.2. C-fibre strength electrical stimuli activate lamina I projection neurons

**Figure 2.**
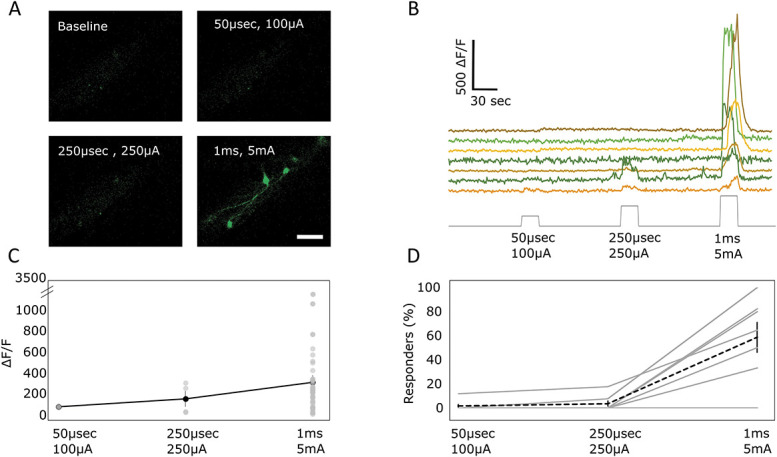
Projection neurons respond to high-intensity electrical stimulation. (A) Example images of projection neurons labelled with calcium indicator, responding to electrical stimulation of the sciatic nerve at increasing intensities. Scale bar = 100 μm. (B) Sample traces of fluorescence intensity changes in projections neurons in response to increasing intensity of sciatic nerve stimulation. (C) Average change in fluorescence intensity of projection neurons responding during electrical stimulation. Each circle represents a responding cell, and the black line represents the average response across all responding cells. The mean of 64 cells across 7 animals ± SEM. (D) Percentage of cells responding to different intensities of electrical stimulation. Percentages of responding cells were calculated relative to all cells visualised postmortem (cf Materials and Methods). Gray lines represent individual mice, and dotted black line represents the average response. The mean of 7 animals ± SEM.

Electrical stimuli were applied directly to the ipsilateral sciatic nerve, through a cuff electrode, at different intensities. Stimulation parameters of 50 µs and 100 µA were used to activate Aβ fibres, 250 µs and 250 µA for Aδ fibres, and 1 ms and 5 mA stimuli were used for suprathreshold stimulation that recruited an additional pool of C fibres. In aggregate, these stimulation parameters were expected to broadly recruit the appropriate populations of nerve fibres. Higher intensity suprathreshold stimuli robustly activated a large subset of lamina I projection neurons (just under 60%) (Movie 1, available at http://links.lww.com/PAIN/B340). Lower-intensity stimuli, in the A-fibre ranges weakly activated a very small subset of lamina I projection neurons (less than 4%) (Fig. 2). It should be noted that there was some variability in the response to electrical stimulation between animals, especially to the A-fibre ranges, where only a subset of animals showed a weak response. This is likely due to the small number of cells recruited and/or small variations in the electrode connectivity.

### 3.3. High-intensity mechanical stimuli activate lamina I projection neurons

**Figure 3.**
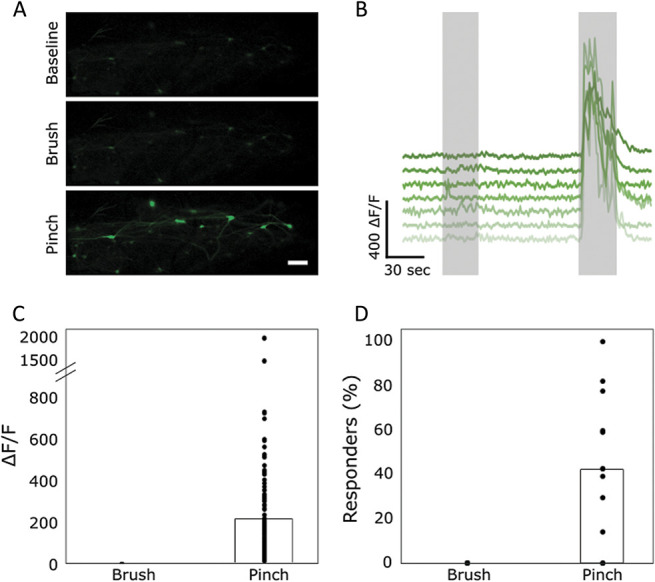
Projection neurons respond to noxious, but not innocuous, mechanical stimulation. (A) Sample images of projection neurons in the dorsal horn responding to mechanical stimulation of the ipsilateral paw. Scale bar = 100 μm. (B) Sample traces of cells responding to mechanical stimulation of the ipsilateral paw. First gray bar represents the duration of the brushing stimulus, and second gray bar represents the duration of the pinching stimulus. (C) Quantification of the fluorescence intensity of cells responding to pinch. None of the cells were found to respond to brush so their ΔF/F is displayed as 0. Data points represent individual cells responding to pinch. The average intensity is displayed by the bar graph. N = 9 animals with n = 127 cells responding to pinch. (D) Percentage of responding cells during brush and pinch of the ipsilateral paw. Data points represent percent of responding cells in each animal, and bar graph represents the average percentage across all animals. Percentages of responding cells were calculated relative to all cells visualised postmortem (cf Materials and Methods). Based on n = 9 animals.

The nociceptive responsiveness of lamina I projection neurons was further confirmed by the application of mechanical stimulation. Innocuous mechanical stimuli (consisting of brushing of the ipsilateral paw) did not activate projection neurons. Noxious mechanical stimuli (pinching of the ipsilateral paw) did activate a large proportion (up to 100% but with high levels of variability, average of 42%) of projection neurons (Fig. 3).

### 3.4. Cold stimuli preferentially activate lamina I projection neurons

**Figure 4.**
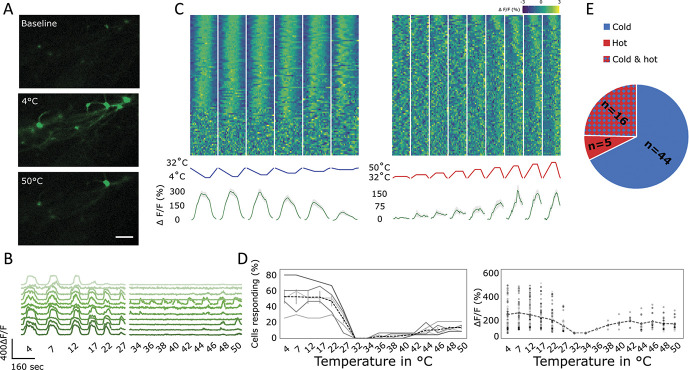
Lamina I projection neuron responses to thermal stimulation. This figure used “simple heating/cooling ramps,” cf Materials and Methods. (A) Representative sample of lamina I projection neurons at baseline and after 4 and 50°C stimulation of the ipsilateral paw. Scale bar = 100 μm. (B) Sample traces of lamina I projection neurons responding to different temperatures applied to the ipsilateral paw. (C) Response of neurons to different intensities of thermal stimuli applied to the ipsilateral paw. Top: Heat map of the response of all recorded lamina I projection neurons, sorted by strength of correlation (in absolute measures) with the respective thermal ramp profile (middle). Bottom: Average trace (green line) of any neuron responding to any cooling or heating ramps. Gray shaded area ± SEM. N = 5 animals and n = 96 cells. (D) Left: Percentage of lamina I projections neurons responding at each thermal stimulus. Continuous lines represent individual experiments. Dashed line represents the average ± SEM. N = 5. Right: Intensity of the response to different types of thermal stimuli. Individual data points represent individual cells and dashed line represents the average. N = 5 animals and n = 96 cells. (E) Distribution of the response of thermally activated cells. Blue: cells responding to cooling but not heating, red: cells responding to heating but not cooling, red/blue chequered: cells responding to both cooling and heating. N = 5 animals, n = 96 cells.

To visualise the response of lamina I projection neurons to thermal stimuli, a Peltier device was placed onto the plantar surface of the ipsilateral paw and its temperature varied at a controlled rate (2°C/s, return at 4°C/s, cf Materials and Methods: “simple heating/cooling ramps”). We found that increases in temperature (“simple heating ramps”) activated fewer neurons compared with decreases in temperature (“simple cooling ramps”, Figs. 4A–E). In addition, responses to heating seemed to predominantly occur in the nociceptive ranges with most responses only being visible above 42°C, with an average threshold temperature of 44 ± 4°C (Figs. 4A–D). Instead responses to cooling were both more pronounced and also evident at innocuous temperatures (Figs. 4A–D). The average threshold for cold responses was 23 ± 5°C, with the largest number of cells having a threshold of 27°C. In response to cooling, both the percentage of cells responding and the intensity of the responses increased with decreasing temperatures with no obvious plateauing until about 4°C (Figs. 4B–D).

In addition, a large percentage of lamina I projection neurons responding to heating of the ipsilateral paw also respond to cooling (“simple heating/cooling ramps”). Indeed, 16 of 21 heat-responding cells also respond to cooling (76%), whereas just 16 of 60 cold-responding cells also respond to heating (27%, Fig. 4E).

Repeated administration of these temperature ramps (“simple heating/cooling ramps”) resulted in a small but nonsignificant decrease in the percentage of responses (Supplementary Fig. 1A, available at http://links.lww.com/PAIN/B339) and had no effect on the intensity of the response (Supplementary Fig. 1B, available at http://links.lww.com/PAIN/B339).

To ensure that the observed responses to heating were not simply a function of the length of the stimulation ramps, we increased the stimulus duration of the “simple heating/cooling ramps” to 30 seconds (Supplementary Figs. 2A–D, available at http://links.lww.com/PAIN/B339). We found that this had no effect on the percentage of cells responding to heating (Supplementary Fig. 2C, available at http://links.lww.com/PAIN/B339) nor the intensity of their response (Supplementary Fig. 2D, available at http://links.lww.com/PAIN/B339) to increases in temperatures. However, we did see a small drop in the percentages of cells responding to decreases in temperature, as compared to the shorter stimulus ramps. This is likely to be related to the fact that stimulus responses decrease when cold temperatures are maintained stable (as will be discussed below, Fig. 4).

#### 3.4.1. Projection neurons are predominantly polymodal, responding to thermal and noxious mechanical stimuli

**Figure 5.**
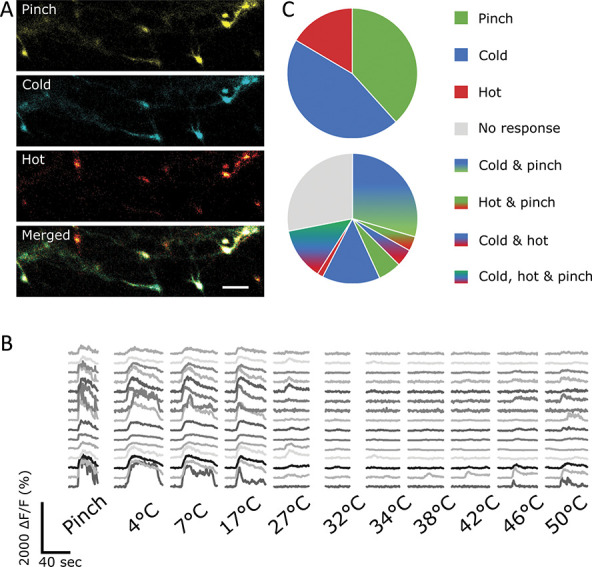
Lamina I projection neurons are predominantly polymodal. (A) Sample images of lamina I projection neurons responding to noxious mechanical (pinching), cooling (4°C), and heating (50°C) stimuli of the ipsilateral paw. (B) Sample traces of neurons responding to various stimuli. (C) Distribution of projection neurons responding to various combinations of different stimuli. Top: Neurons responding to pinching (green), cooling (blue), and heating (red) (included are cells that respond unimodally or polymodally to these stimuli). Bottom: distribution of projection neurons responding only to pinching (green), cooling (blue), heating (red), a combination of these stimuli (mixed colours), or nothing (gray).

To understand the response profiles of lamina I projection neurons in more detail, we applied mechanical and thermal stimuli. The mechanical stimulus was a series of pinches, and the thermal stimuli used were the “simple heating/cooling ramps.” Both stimulus modalities are described in Materials and Methods and were applied successively in the same experiment, allowing us to assess the level of polymodality in this population of cells (Fig. 5, Movie 2, available at http://links.lww.com/PAIN/B341). We found that the largest subset of responsive cells was activated by both pinch and cold (41 of 139 cells; 29%), whereas the second largest group combined heat, cold, and pinch responses (18 of 139 cells; 13%) (Fig. 5c). All unimodal responses (pinch, cold, or heat alone) combined to only 30 of 139 recorded cells (22%), with cold responses making up the largest proportion (20 unimodal cold-responding cells among 30 total unimodal cells; 67%). This resulted in 59 of 72 mechanosensitive cells also responding to cold stimuli (82%) and 23 of 72 mechanosensitive cells also responding to heating (32%). Of these polymodal mechanosensitive cells, 18 of 72 cells (25%) were polymodal for both thermal modalities. In these experiments, data from both longer and shorter “simple heating/cooling ramps” were included.

However, it should be noted that the level of polymodality between mechanical and thermal responses should be considered an estimate of the minimum. Despite all efforts to ensure stimulation of identical receptive fields, there is no guarantee that the same breadth and depth of stimulation of the paw was achieved for these different stimulus modalities.

#### 3.4.2. Cold responses decrease during a stable cold stimulus, whereas heat responses remain stable

**Figure 6.**
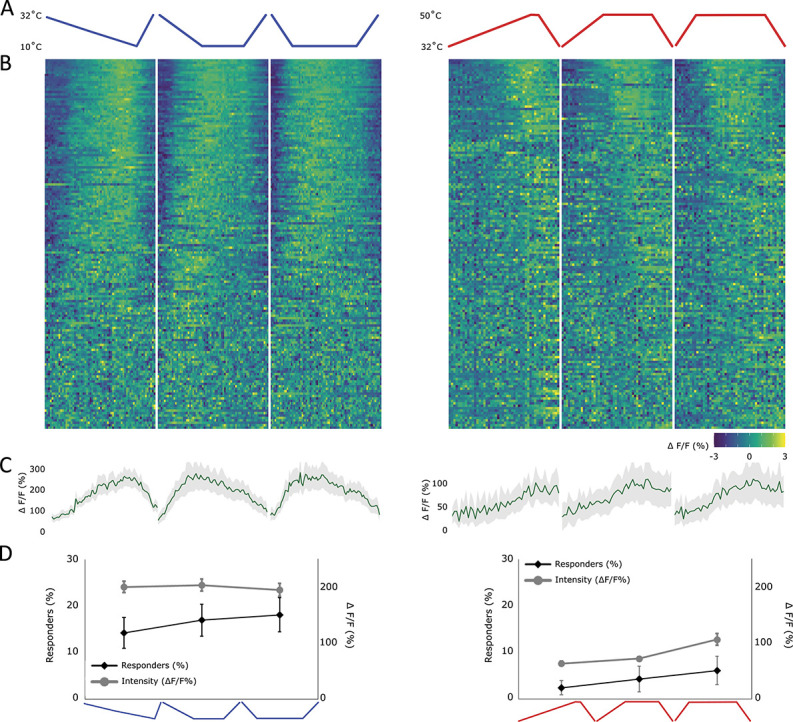
Responses of lamina I projection neurons to temperature ramps of different speeds. This figure used “rate of change heating/cooling ramps,” cf Materials and Methods. (A) Profile of the cooling (blue) and heating (red) stimulus applied to the ipsilateral paw, including a slow (0.2°C/s), medium (0.5°C/s), and fast ramp (2°C/s) (left to right). (B) Heat map of the response of all recorded lamina I projection neurons, sorted by strength of correlation with the respective thermal ramp profile (in absolute measures). Each cell's fluorescence is normalised (mean subtracted and divided by SD). Left: neurons responding to the above depicted cold ramps and right: neurons responding to the above depicted hot ramps. (C) Average profile of cells showing any response to either a cold (left) or a hot stimulus (right). Gray shaded area ± SEM. (D) Percentage of cells responding (diamond shapes, black line, left y-axis) and the average intensity of the response (circles, gray line, right y-axis) to each ramp individually. Mean ± SEM. N = 7 animals and n = 152 cells.

To understand the response profiles of this group of cells in more detail, a series of ramps with different speeds were applied to the ipsilateral paw (Fig. 6A, “rate of change heating/cooling ramps,” cf Materials and Methods). The total time of the stimulus was maintained constant, and those with a faster ramp therefore had longer periods of a stable end temperature (10°C for cold and 50°C for hot). In the case of cold ramps (“rate of change cooling ramps”), the fluorescence intensity increased to similar levels during the phase of decreasing temperature in all 3 ramps, but the peak was reached at different speeds, in line with the temperature (Figs. 6A–C). The rate of change did not seem to affect the response intensity of these neurons because the responses seemed to closely follow the temperature intensity of the on-ramp (Figs. 6A–C). This contrasts with the psychophysical percept of cold that does seem to depend on the rate of change.^22^ However, the stable phase of the ramp was associated with a reduction in the fluorescence intensity of lamina I projection neurons. Therefore, the fluorescence intensity during this period decreased most in the fast ramp, compared with the slower ramps (Figs. 6A–C). Thus overall, these responses seemed to not only encode the absolute skin temperature applied during these 3 “rate of change cooling ramps” but also showed accommodation to a maintained temperature.

Although cooling responses clearly declined when a stable end temperature was reached, this pattern was not observed during peripheral heating (“rate of change heating ramps”). Instead responses to heating seemed to be determined by a noxious threshold and maintained for the duration of that temperature (Figs. 6A–C). Again, the rate of change had no noticeable effect on the response intensity in lamina I projection neurons. However, the percentage of responding cells, although smaller compared with cold-responsive cells, increased from 2% to 6% from the slowest to the fastest ramping speed (Fig. 6D). This could be due to a variation in the rate of change but is more likely due to the prolonged period of hot stimulation after faster ramps. Overall therefore, this system seems to encode absolute temperature within the noxious ranges and maintains a stable response during a stable, noxious temperature.

#### 3.4.3. Cooling responses are mostly determined by end temperature, but heating responses show an unexpected sensitivity to adaptation temperatures

**Figure 7.**
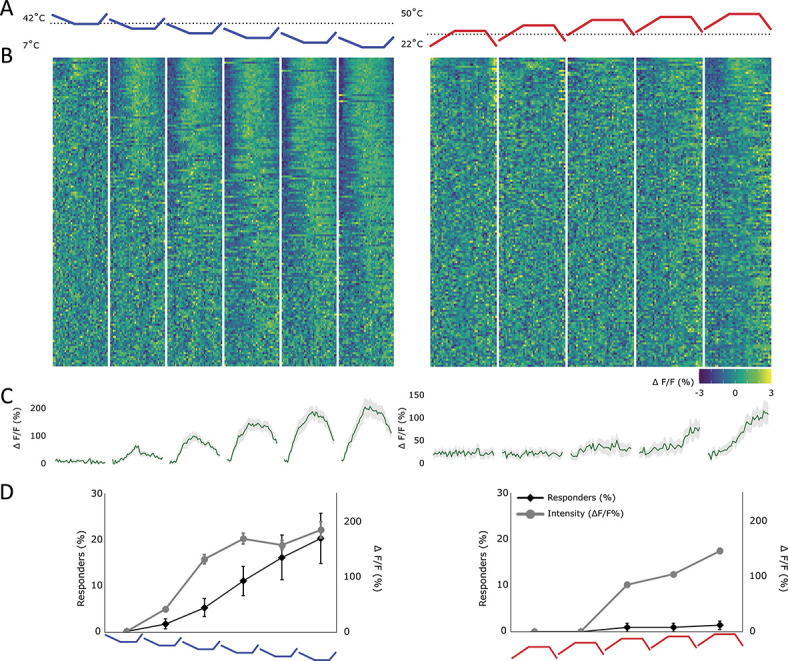
Response of lamina I projection neurons to ramps with variable end temperatures. This figure used “baseline variable heating/cooling ramps,” cf Materials and Methods. (A) Profile of the cold (blue) and hot (red) stimulus applied to the ipsilateral paw. The end temperatures were varied (for cold: 7-32°C and for heat: 35-50°C), but the amount of change remained stable (for cold: 10°C and for hot: 13°C). Black dotted line represents the normal skin temperature (32°C). (B) Heat map of the response of all recorded lamina I projection neurons, sorted by strength of correlation with the respective thermal ramp profile (in absolute measures). Each cell's fluorescence is normalised (mean subtracted and divided by SD). Left: neurons responding to the above depicted cold ramps and right: neurons responding to the above depicted hot ramps. (C) Average profile of cells showing any response to either a cold (left) or a hot (right) stimulus. Gray shaded area ± SEM. (D) Percentage of cells responding (diamond shapes, black line, left y-axis) and the average intensity of the response (circles, gray line, right y-axis) to each ramp individually. Mean ± SEM. N = 7 animals and n = 152 cells.

To understand the response of lamina I projection neurons to the amount of change vs the end temperature, a series of thermal ramps were applied to the ipsilateral paw. The first of these ramps (“baseline variable heating/cooling ramps,” cf Materials and Methods) varied both the end temperature (between 7 and 32°C for cold and 35 and 50°C for heat) and the baseline temperature (between 17 and 42°C for cold and 22 and 37°C for heat) while maintaining a stable amount of change in each ramp (10°C for cold and 13°C for heat). The baseline temperature was maintained for 2 minutes before the onset of the ramp. Responses to both heating and cooling seemed linked to the end temperature and unresponsive to the magnitude of change (Figs. 7A–D). However, cooling responses to the “baseline variable cooling ramps,” as seen before (refer to Fig. 4, using “simple cooling ramps”), were evident already at innocuous temperatures (Figs. 7A–D). Indeed, the only decline in temperature that did not elicit a response from lamina I projection neurons was a response entirely contained above skin temperature (Figs. 7A–D).

Despite the lower baseline temperatures being well within the response range of the cooling cells, their adaptation to maintained cold temperature (Fig. 6) meant that responses to each ramp originated from a near zero response to reach a peak determined by the end temperature. It should be noted that across the ranges of cells studied, a small number of cells did show an increase in responsiveness to lower baseline temperatures; however, these were few enough to have no effect on the aggregate response and seemed to have no obvious other defining characteristics.

As hot responsive cells generally had a noxious threshold, a baseline temperature of a maximum of 37°C unsurprisingly did not lead to an activation of these cells (using “baseline variable heating ramps”).

Overall, the results from the “baseline variable heating/cooling ramps” suggest that cold responses can adapt to variable baseline temperatures and respond to decreases in temperature with a threshold just above the resting skin temperature of 32°C. In addition, the end response magnitude and the recruitment of cold responses seem determined by the absolute temperature being reached, irrespective of the amount of change. Hot responses, although small in magnitude and number, seem to be contained almost entirely within the noxious ranges.

**Figure 8.**
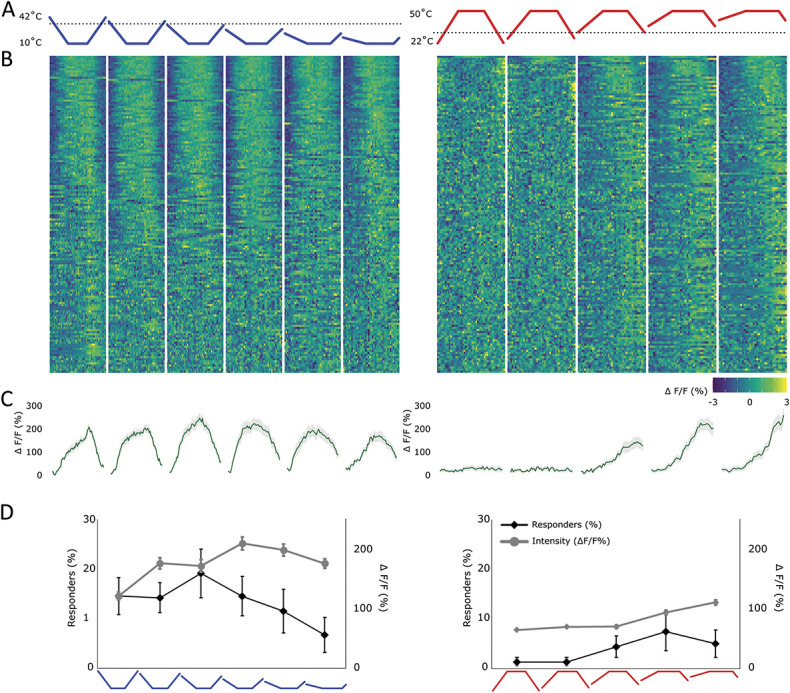
Response of lamina I projection neurons to ramps with variable amounts of change. This figure used “end temperature stable heating/cooling ramps,” cf Materials and Methods. (A) Profile of the cold (blue) and hot (red) stimulus applied to the ipsilateral paw. Black dotted line represents the normal skin temperature (32°C). End temperatures were maintained stable (10°C for cold and 50°C for heat), but the baseline temperature was varied (between 42-17°C for cold and 22-42°C for heat). (B) Heat map of the response of all recorded lamina I projection neurons, sorted by strength of correlation with the respective thermal ramp profile (in absolute measures). Each cell's fluorescence is normalised (mean subtracted and divided by SD). Left: neurons responding to the above depicted cold ramps and right: neurons responding to the above depicted hot ramps. (C) Average profile of cells showing any response to either a cold (left) or a hot stimulus (right). Gray shaded area ± SEM. (D) Percentage of cells responding (diamond shapes, black line, left y-axis) and the average intensity of the response (circles, gray line, right y-axis) to each ramp individually. Mean ± SEM. N = 7 animals and n = 152 cells.

A further set of ramps (“end temperature stable heating/cooling ramps,” cf Materials and Methods) maintained a stable end temperature (10°C for cold and 50°C for heat) but had variable baselines (between 42-17°C for cold and 22-42°C for heat). This resulted in a variation in the amount of change the periphery was exposed to, while maintaining a stable end temperature (Fig. 8A). The intensity of cooling responses (“end temperature stable cooling ramps”) seemed to be relatively invariant to the baseline temperature and thereby the amount of change (Figs. 8B–D). Indeed, despite a large variation in the start and end temperature, the responses to decreases in temperature seemed consistent across all ramps, starting and returning to near resting values, even when the baseline was well within the range of response. Yet again, it seems that certain mechanisms lead cold-responding cells to be insensitive to the amount of change while nevertheless adapting to prolonged cold stimuli. Whether these mechanisms originate within this population itself or from first-order cells or indeed supraspinal mechanisms is yet unknown.

The responses to heating (“end temperature stable heating ramps”) on the other hand seemed strongly dependent on the baseline temperature (and therefore the amount of change), despite the stable end temperature. The intensity of the response recorded from lamina I projection neurons seemed to increase for higher baselines (Figs. 8B–D). This suggests that maintenance of the periphery at a progressively higher temperature for a period before the ramp affected projection neurons' heat responses and resulted in increased responsiveness.

Large and fast increases in temperature were expected to result in significant activation of the nociceptive lamina I projection neuron system, but interestingly, if the baseline was kept below skin temperature (the first 2 ramps in the “end temperature stable heating ramps” protocol), the response of projection neurons was completely abolished, despite the peripheral stimulus reaching 50°C (Figs. 8B–D).

An overview considering all ramps applied suggests that cold-responsive projection neurons respond in their majority to innocuous cold temperature ranges (mostly showing a threshold between 22 and 27°C) and noticeably adapt to stable temperatures, maintained for more than a few seconds. They also seem to encode the intensity of the end temperature while being invariant to the baseline/adaptation temperature. That is, even if a ramp starts/finishes well within their response ranges, any decline in temperature results in an almost full decline of the response back to baseline.

Hot responses in turn seem to be confined to the noxious ranges (mostly exhibiting a threshold above 44°C) in which responses are maintained as long as the stimulus is present, with little adaptation. Interestingly, hot responses also seemed to be affected by the baseline/adaptation temperature, even if this temperature was not within the response range of these cells. Thus, warmer baseline temperatures seemed to prime the response of projection neurons responding to noxious heating, whereas baseline temperatures below 32°C (considered skin temperature) seemed to completely abolish any responses, even to noxious temperatures, suggesting an interaction between the processing of cold and hot cutaneous temperatures.

## 4. Discussion

Using standard single-photon microscopy together with a genetically encoded calcium indicator (GCaMP6s), we were able to study the function of spinoparabrachial projection neurons in lamina I of the mouse spinal cord in vivo. The stability of the preparation was sufficient to visualise these cells over several hours while applying multiple peripheral stimuli. The use of a single-photon microscope allowed us to increase the available signal and signal depth by opening the pinhole. This enabled greater signal and stability over time (with small movements now being contained within the depth of available signal) and reduced the effect of the curvature in the spinal cord. As these cells also tend to be in a single, nonoverlapping plexus, the increase in signal from out-of-focus areas resulted in few drawbacks.

It is known that 85% to 95% of lamina I projection neurons project to the lateral parabrachial nucleus in the rat^44,49,53^ and likely a similarly high proportion in the mouse.^5^ Therefore, we were able to label a representative plexus of lamina I projection neurons with a single injection of AAV9 expressing GCaMP6s into the lateral parabrachial area.

Using this approach, we were able to confirm the noxious responsiveness of lamina I spinoparabrachial neurons after electrical and mechanical stimuli.^2–4,29,38,55,56^ However, it should be noted that the percentage of lamina I spinoparabrachial projection neurons responding to noxious mechanical stimuli was variable. One of the major benefits of in vivo imaging of projection neurons is the ability to visualise an entire network of cells simultaneously. However, this also requires the simultaneous stimulation of multiple receptive fields, which is significantly less controllable compared with the stimulation of single receptive fields, as is performed in electrophysiological experiments. As a result, a greater variability in the number of cells recruited by, eg, mechanical stimuli is to be expected.

In addition, in line with previous reports we found that most mechanically sensitive cells were also polymodal for either heating or cooling of the plantar surface of the same paw.^4^ In addition to finding a large proportion of lamina I projection neurons that were responsive to only noxious levels of mechanical stimuli, we found a group of cells responding to heating of the ipsilateral paw. These responses were also dominated by noxious inputs, and although the percentage of cells responding to noxious heating was lower than in some other reports,^2,4^ it is broadly in line with other studies.^20^

Overall, we found that lamina I spinoparabrachial neurons mostly respond only within the noxious ranges for mechanical and heating stimuli and that the response profiles of these neurons are predominantly polymodal. These findings are broadly in line with previous reports of subsets of lamina I neurons in general^7,8,11,32,35,41,50^ and projection neurons in particular.^2–4,16,17,24,29,33,34,62^ Such overall concordance between our findings and those of previous electrophysiological investigations provides confidence in the use of calcium as a proxy for activity.

Our findings also highlighted a strong sensitivity to innocuous cooling. Although previous reports have noted the presence of a class of cold-responsive neurons both in parabrachial and thalamic projection neurons,^2,17,18,20,21,34,56^ our findings are notable in the following key aspects: cold responses seemed to be very common in this plexus of cells with 61% of cells responding to cooling; we found innocuous cold responses to be extremely common with 82% of cold-responsive neurons having response thresholds between 22 and 27°C, well within the innocuous ranges^9,22,58,63^; and we found that the majority (76%) of neurons responding to cooling were also polymodal. However, a small proportion (14% of all neurons) seemed to respond exclusively to cooling of the ipsilateral paw, perhaps representing a labelled line for cold stimuli.^15,17,20,21,40^

The overwhelming response of lamina I projection neurons to cooling was not expected. Our own work on the peripheral nervous system using an identical stimulation paradigm,^10^ as well as others' work^19,26,36,59^ has shown that cold sensitive neurons are a relatively rare population among primary afferents. For one of the main spinal cord output systems to be so heavily dominated by a marginally represented peripheral neuronal population was not only unexpected but also poses significant questions as to the circuitry underlying this specialised relay system. In addition to finding a strong dominance for cold responses in lamina I projection neurons, we were able to characterise the thermal responses of these cells in response to a variety of thermal ramps. These ramps included test stimuli for the sensitivity of projection neurons to overall temperature (Fig. 4), the sensitivity to the rate of change (Fig. 6), the sensitivity to the amount of change (Figs. 7–8), and the sensitivity to the baseline or adaptation temperature (Figs. 7–8).

Overall, the intensity of the responses to decreases in temperature was mostly monotonic with a plateauing of response intensities below 7°C. Despite this sensitivity and ability to encode decreases in temperature, responses declined when a stable temperature was reached. Despite this adaptation to stable temperatures, response intensities were determined predominantly by the end temperature.

Responses to heating, elicited in many of the same cells that also respond to cooling, were predominantly in the noxious ranges and were fewer in number and lower in intensity compared with cooling responses. These cells did not seem to encode increases in temperature within the noxious ranges well, with response intensities plateauing, in aggregate, between 42 and 44°C. In addition, hot responses seemed to remain stable in response to noxious temperatures, with little adaptation. However, unexpectedly, responses to heating showed a strong dependence on baseline or adaptation temperatures with higher baseline temperatures resulting in greater response amplitudes, despite these temperatures being below the noxious range and thereby mostly below the response threshold.

A previous study, using a similar technique to that described here, namely in vivo imaging of unidentified spinal cord neurons, was also able to activate thermally responsive spinal cord neurons in the superficial layers of the dorsal horn.^45^ However, in contrast to our findings they showed a predominance of heat responses over responses to cooling. In addition, they concluded that cold responses were sensitive to change rather than absolute temperatures, whereas heating responses were only driven by the absolute end temperatures, rather than the change in temperature.^45^ These discrepancies between our results are likely to result from the population of cells being studied. While Ran et al.^45^ injected a calcium indicator into the dorsal horn of the spinal cord, thereby labelling all available cells nondiscriminately, we chose to focus on the subset of lamina I spinoparabrachial projection neurons, which make up only 5% of the total pool of neurons in the mouse dorsal horn.^5^ It is likely therefore that Ran et al. were predominantly looking at response characteristics of interneurons, whereas we studied the output system of the spinal cord, the projection neurons. However, a few key aspects seem to remain consistent between these results, namely the dominance of noxious thresholds in hot responses and the adaptation of responses to prolonged cold stimuli.

We here report on a system that is sensitive to noxious mechanical and heating stimuli and that has been implicated in the development of chronic pain.^37,43^ In addition, we find a striking sensitivity of this system to cooling, raising the question whether this plexus of lamina I projection neurons may play a key role in cold allodynia and hypersensitivity often seen in neuropathic pain states (for review refer to Ref. 27). In support, projection neurons (albeit spinothalamic projection neurons in the cat) have already been implicated in the paradoxical “thermal grill” illusion^14^ in which adjacent innocuous warm and cold stimuli result in a sensation of strong, often burning heat.

The heavy impingement of cold stimuli on the responses of lamina I projection neurons could also suggest a role for them in homeostatic mechanisms. The parabrachial area, the projection target of cells studied here, has been implicated in the thermostatic control of body temperature.^31,42,64^ For a system to be involved in homeostatic temperature control it is likely to require information (1) across a wide thermal range, (2) sensitive to absolute levels of temperature, and (3) sensitive to any perturbations of the temperature. Our findings suggest that lamina I projection neurons could provide a combination of this information to the lateral parabrachial area: This system is not only dominated by cold responses, but these responses seem to be highly sensitive even to small decreases in temperature, while simultaneously encoding information across the innocuous and noxious ranges. Indeed, the ability of this system to adapt to stable temperatures could enhance its sensitivity to further changes in temperature, across a wide range (from innocuous to noxious). Such adaptation is not uncommon in the sensory system and is likely to enhance our ability to detect changes across a wide range of senses.^12,28,46,47,57,60^

## Conflict of interest statement

The authors have no conflicts of interest to declare.

## Appendix A. Supplemental digital content

Supplemental digital content associated with this article can be found online at http://links.lww.com/PAIN/B340, http://links.lww.com/PAIN/B339, and http://links.lww.com/PAIN/B341.

## Supplemental video content

A video abstract associated with this article can be found at http://links.lww.com/PAIN/B342.

## Supplementary Material

SUPPLEMENTARY MATERIAL
