# Supplementary figures and images for: Encoding of cutaneous stimuli by lamina I projection neurons
Source: Pain. 2021 Mar 24;162(9):2405–17. doi: 10.1097/j.pain.0000000000002226 (PMC8374708; doi:10.1097/j.pain.0000000000002226)

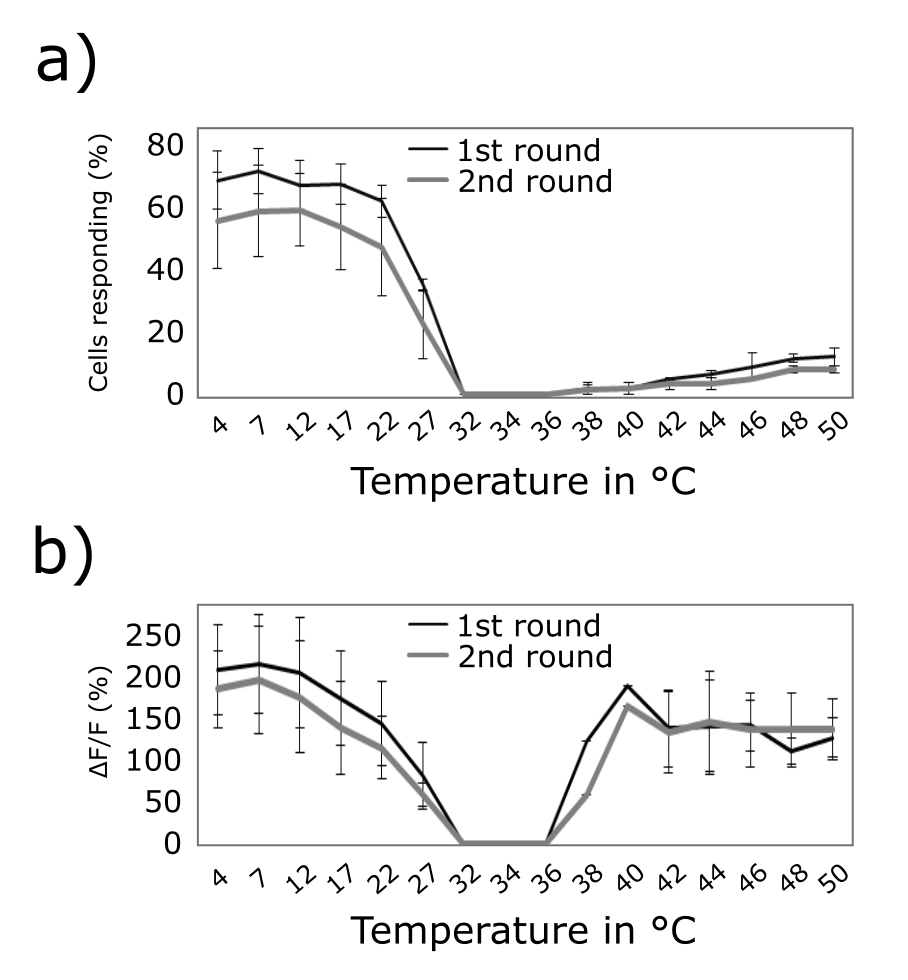

Supplement: SUPPLEMENTARY MATERIAL [file jop-162-2405-s001.png]

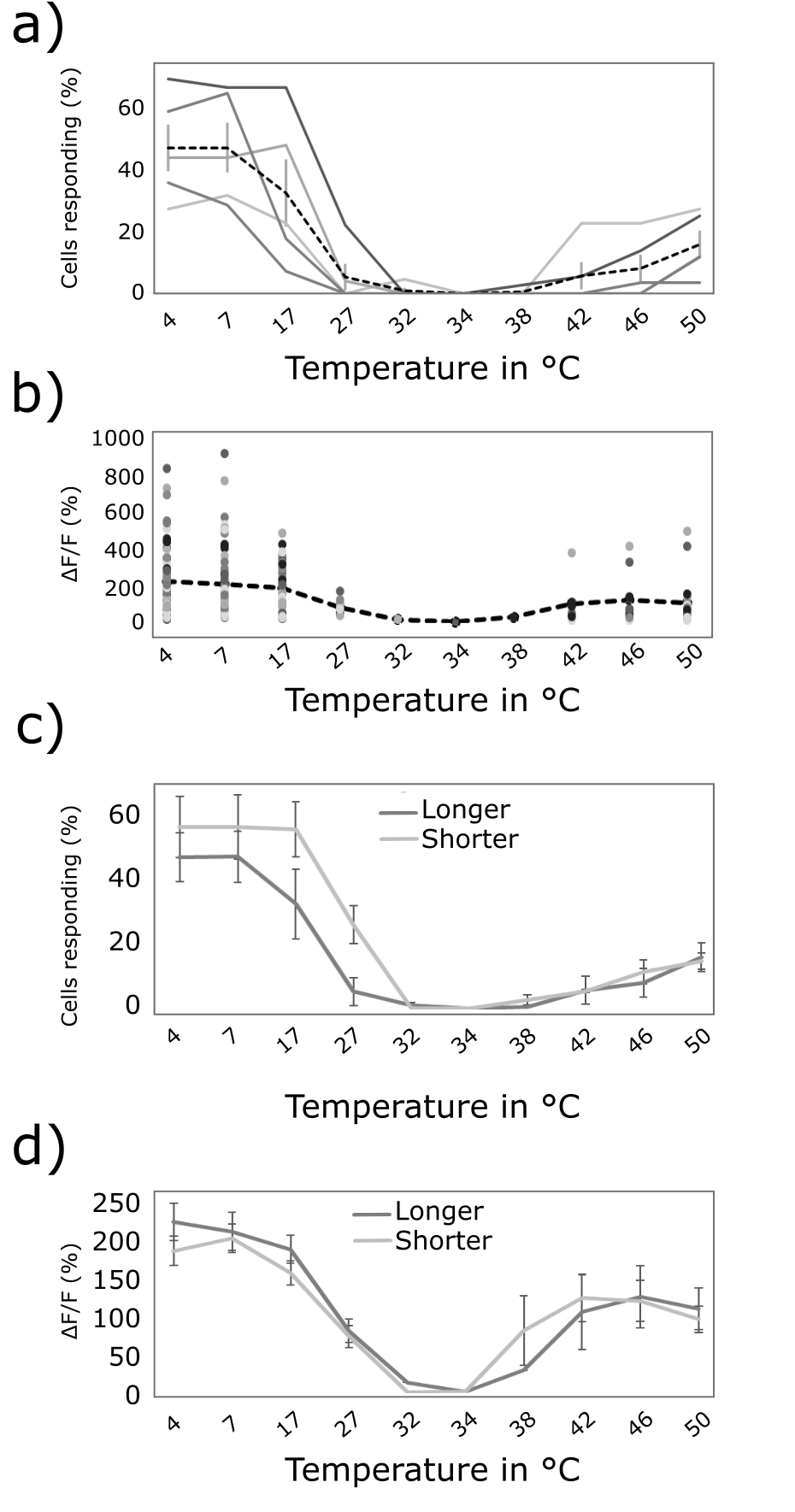

Supplement: SUPPLEMENTARY MATERIAL [file jop-162-2405-s002.png]
